# Adjuvant therapy by high-speed burr may cause intraoperative bone tumor seeding: an animal study

**DOI:** 10.1186/s12891-020-03544-3

**Published:** 2020-07-31

**Authors:** Pai-Han Wang, Chia-Lun Wu, Chao-Ming Chen, Jir-You Wang, Po-Kuei Wu, Wei-Ming Chen

**Affiliations:** 1 Department of Orthopaedics, Therapeutical and Research Center of Musculoskeletal Tumor, Taipei Veterans General Hospital, Taipei, Taiwan; 2 Department of Orthopaedics & Traumatology, Taipei Veterans General Hospital, 201 Shi-Pai Road, Beitou District, Taipei, 11217 Taiwan; 3 Department of Orthopedics, School of Medicine, National Yang-Ming University, Taipei, Taiwan; 4 Institute of Clinical Medicine, School of Medicine, National Yang-Ming University, Taipei, Taiwan; 5 Institute of Traditional Medicine, School of Medicine, National Yang-Ming University, Taipei, Taiwan

**Keywords:** Giant cell tumor, Polymethyl methacrylate, Local recurrence, Intralesional curettage, Iatrogenic

## Abstract

**Background:**

Bone tumors are often treated with intralesional curettage. High-speed burring, an adjuvant therapy, was performed to maximize the tumor cell killing; however, tumor recurrence might still occur, which may be caused by residual tumor or local tumor spread during surgery.

**Methods:**

A porcine cadaver (femur) was utilized to determine whether the use of a high-speed burr causes bone cement spray. To mimic residual tumor after curettage, luminescent cement was smeared on two locations of the bone cavity, the wall and the bottom. The cavity in the femoral bone was then placed in the middle of a sheet of drawing paper featuring 10 cm, 20 cm, and 30 cm concentric circles. The luminescent cement was then burred totally with a high-speed burr.

**Results:**

The intensity of the area in the wall in circle I was 72.6% ± 5.8%; within circle II, it was 22.1% ± 4.2%; and within circle III, it was 5.4% ± 1.5%. The intensity of the area within the bottom of the femoral bone within circle I was 66.5% ± 6.1%, within circle II was 28.1 ± 4.8%, and within circle III, it was 5.4% ± 1.4%. The amount of luminescent cement seeding decreased with distance, but there was no difference while burring at different locations of the bone cavity. Under the handpiece cover, a greater amount of cement spray was retained in circle I during burring of the cement in the bottom of the cavity and less was sprayed out in circle III.

**Conclusions:**

High-speed burring may cause explosive bone cement spray, which could extend to 20 cm. The intensities of spray did not decrease, even when the handpiece cover was used. The wide range of bone cement spray caused by high-speed burr was inspected in this pilot study, which may lead to tumor seeding.

**Level of evidence:**

Level IV, therapeutic study. See Guidelines for Authors for a complete description of levels of evidence.

## Background

Bone tumors are a heterogeneous group of diseases with different biological characteristics that variably alter the appearance, architecture, and inherent stability of the skeleton. The benign bone tumors, such as giant cell tumor (GCT), chondroblastoma, or fibrous dysplasia, are often managed by intralesional curettage and burring. While, benign aggressive and primary low-grade malignant tumors generally require surgery to obtain local control [1]. To achieve maximum tumor resection, adjuvant therapy with a high-speed burr after tumor excision is frequently performed to manage bone tumors, such as aneurysm bone cyst, GCT, and low-grade chondrosarcoma [2–4].

GCT of bone is one of the benign neoplasms that has the potential to recur or to lead to pulmonary metastasis. GCT accounts for approximately 5% of all primary bone tumors and typically occurs around the knee. In large studies, the recurrence rate of GCT after surgery varies from 4 to 30% [5]. To reduce the local recurrence rate, different adjuvant therapies are performed after tumor curettage [4]. To preserve the greatest function of the limb, the goal of adjuvant therapies is to extend the tumor excision zone several millimeters beyond the limit of the mechanical curettage [6].

Surgery is performed by creating an adequate cortical window, allowing for visualization of the lesion and curettage of the grossly visible portion of the tumor. Several types of adjuvant therapies, including phenol/alcohol, and cryotherapy, have been performed to maximize tumor kill. Meanwhile, the mechanical adjunct of a high-speed burr is performed to extend the margins of the tumor resection [4]. Use of a high-speed burr may also preserve the joint adjacent to the tumor [7].

Higher contamination rate was found while examining airborne bone chips after use of a high-speed burr; the chips struck nonsterile surfaces before landing on the surgical site [8]. In order to extend the margins of the tumor resection, the osseous architecture could be weakened, which may lead to intraoperative cortical penetration and postoperative fracture. Compared with other types of adjuvant therapy, adjuvant therapy by high-speed burr alone had no effect on the recurrence rate [9]. When burring, the bone spray can be regarded as the tumor-bearing bone particle. We conducted experiments using a porcine cadaver to determine: (1) the amount of tumor-bearing bone seeding, and (2) the range over the surgical area and surgical glove of tumor-bearing bone due to use of the high-speed burr and use of the burr with a handpiece cover.

## Methods

### Luminescent bone cement synthesized

First, the luminescent bone cement represents the tumor-bearing bone particle during high-speed burring. The cadaver of three 6-month-old, 120-kg pigs, the Landrace/Yorkshire sow, obtained from National Laboratory Animal Center was utilized during the experiments. A 4.0-cm-long, 2.0-cm-wide, and 3.0-cm- deep cavity was created with a handheld electric drill on the medial epicondyle of a femur of the pig. The residual trabecular bone was removed by handheld electric burr, to ensure that the volume of cavity contained 24.0 mL of water. Second, in order to record the bone cement spray in a darkroom, the cement was mixed with 2.0 g of luminescent powder (First Chemical, Taipei, Taiwan), The luminescent bone cement was then synthesized by mixing 2.0 g of luminescence powder, and 10.0 g of powder component (7.5 g of methyl methacrylate-styrene copolymer, which contained 1.7% benzoyl peroxide, 1.5 g of polymethyl methacrylate, and 1.0 g of barium sulfate). Finally, this was mixed with 5.0 mL of liquid (4.875 mL of methyl methacrylate monomer, 0.125 mL of N, N-dimethyl-p toluidine, and 0.375 mg hydroquinone) of radiopaque bone cement (Simplex™ P Radiopaque Bone Cement, Stryker, Mahwah, NJ, USA). The luminescent bone cement was stirred continuously until it formed a dough-like mass. The mass was divided into three equal parts (5.5–5.7 g), smeared on the bottom of the cavity, and kept in a laminar flow hood for 30 min at room temperature. The second step was repeated by smearing the material to the side wall of the cavity. The thickness of the cement layer smeared on the bottom and to the side wall was 3 mm ± 0.3 mm. There is no difference between the thickness on the bottom and the side wall.

### Bone cement burred and data recorded

The cavity of the femoral bone was placed upon and operated on while in the center of a piece of drawing paper containing 10 cm, 20 cm, and 30 cm concentric circles. The luminescent bone cement was burred with a 2.2-mm high-speed burr at 70,000 rpm (Conmed Linvatec PowerPro PRO2000™, Largo, FL, USA). The size of the burr was chosen according to the size of the bone of porcine and the speed of the burr was the maximum to illustrate the most influence of the burr. In the darkroom, the splashed luminescent bone cement specimen was exposed under 365 nm UV light for 5 min and photographed with a 10-s exposure using a Canon 550D camera (Canon, Tokyo, JP). The luminescent intensity (signal divided by the area) of circle I (< 10 cm), circle II (≥ 10 and < 20 cm), and circle III (≥ 20 and < 30 cm) were analyzed with ImageJ processing software (National Institutes of Health, Bethesda, MD, USA). Since we only discuss the specimen during surgery, data for areas > 30 cm were ignored.

**Fig. 1:**
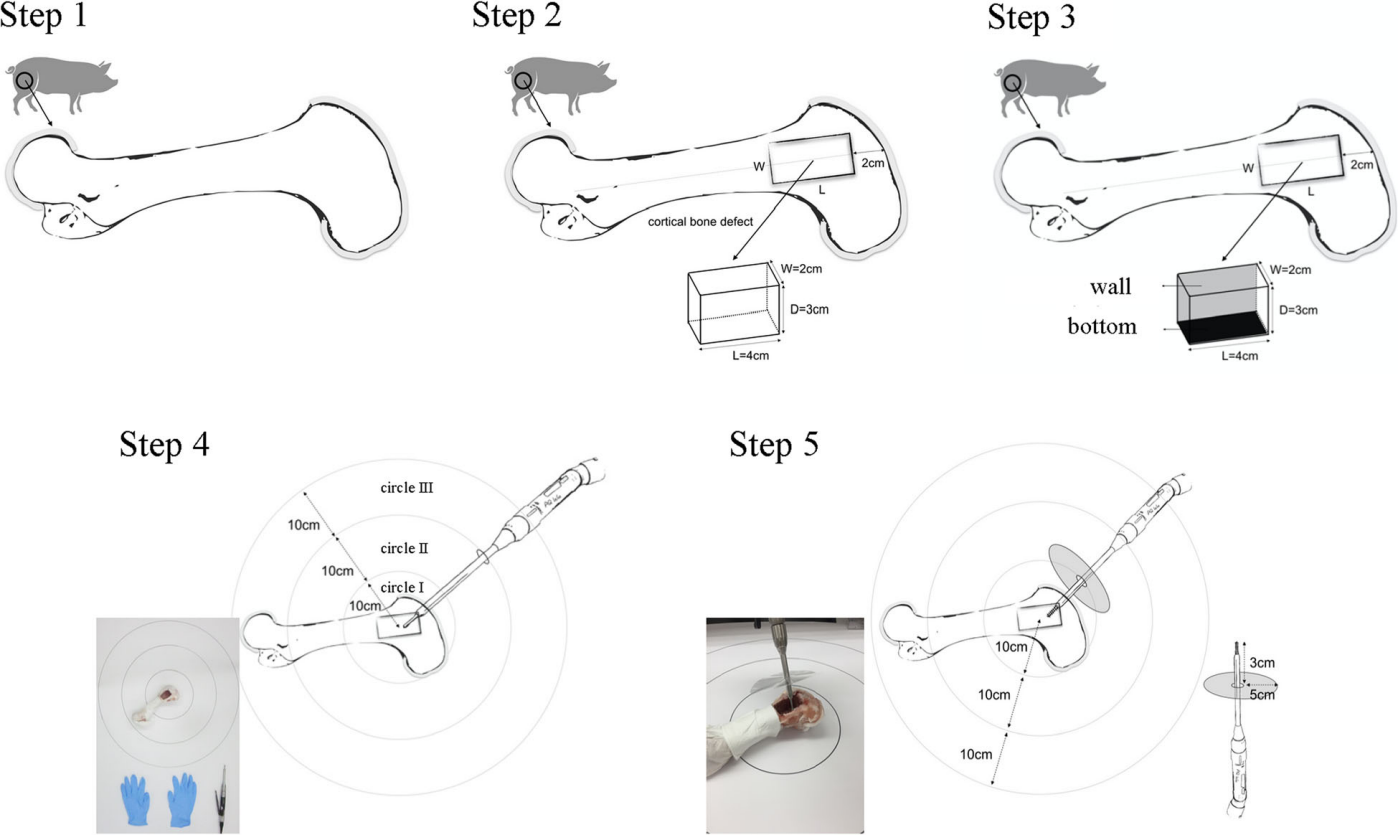
The steps of the study. Step 1, a femoral medial epicondyle from the pig. Step 2, A created cavity Step 3, cement was bound to the wall and the bottom. Step 4, the cement was burred. Step 5, burred with handpiece coverage

The second part of the study involved recording the amount of splashed luminescent bone cement under the handpiece cover. Prior to the test, we had designed a handpiece cover to evaluate the efficiency of a cover to prevent luminescent bone cement spray. The handpiece cover was made with a 5-cm diameter piece of plastic cut from an autoclave bag and placed 3 cm away from the head of the burr. The luminescent bone cement was burred and analyzed as the first part of the experiment (Fig. 1).

The third part of the study involved recording the amount of luminescent bone cement splashed on the surgical glove. The dorsal sides of a pair of rubber gloves were glued together to enhance the connection with bone cement. Then, luminescent bone cement splashed over the glove was exposed under 365 nm UV light for 5 min, and then exposed for 10 s longer, once again recorded with the Canon 550D camera in the darkroom. The cement on the glove was recorded with and without the handpiece cover. The animal use in this article has been reviewed and approved by the Institutional Animal Care and Use Committee (IACUC) and the IRB of the authors’ affiliated institutions.

### Statistical analysis

Data were shown as mean ± standard deviation. Student t-test and one-way ANOVA were used to analyze the splashed area of bone cement.

## Results

### Bone cement without cover

**Fig. 2:**
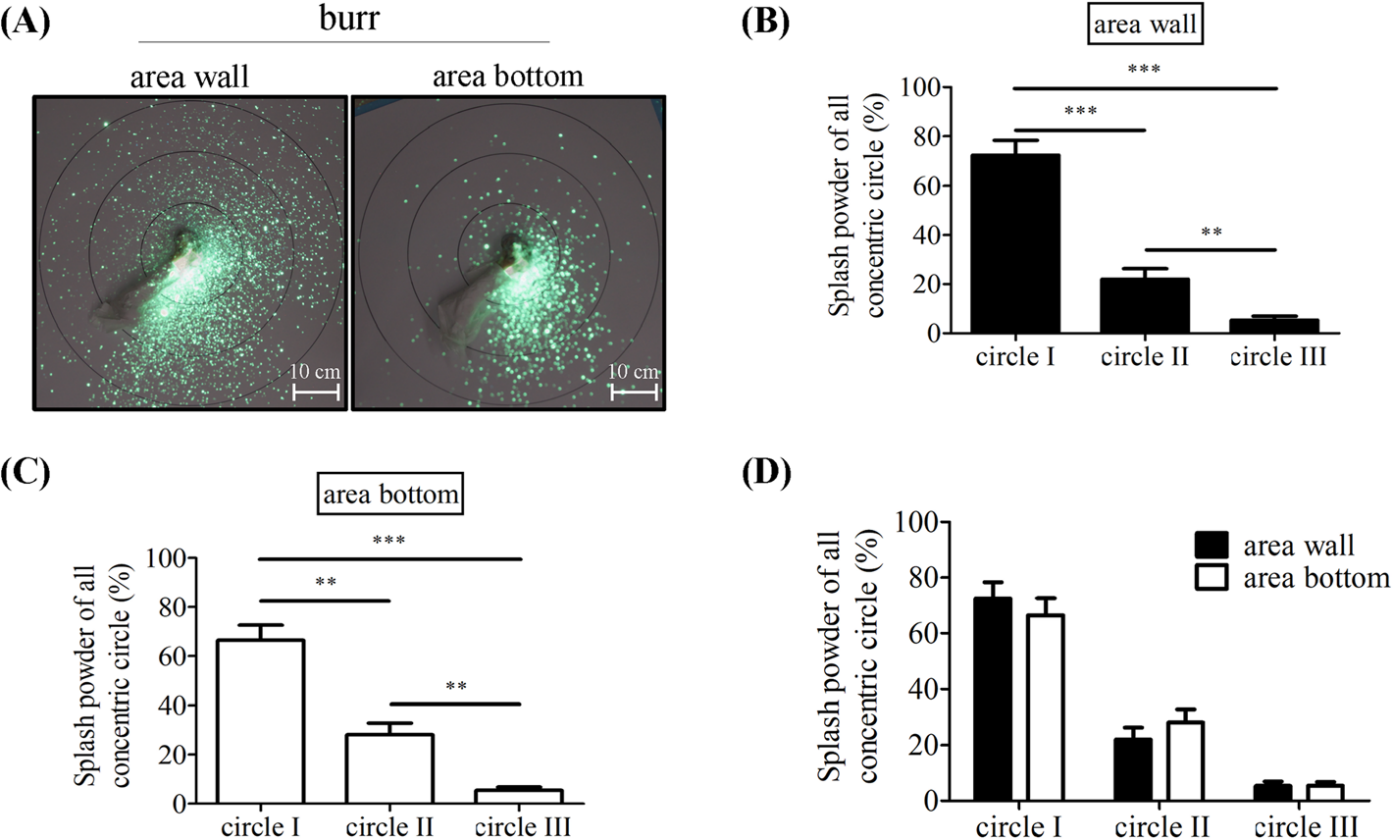
Bone cement spray without cover. **a** Luminescent bone cement splash after burring the bone cement without cover. **b**-**d** Histogram of luminescent bone cement splash on circles I, II, and III after burring the area wall and bottom. ***, *p* < 0.001, **, *p* = 0.003

The splashed luminescent bone cement was exposed under 365 nm UV light for 5 min, with a continuous 10-s exposure, recorded by the Canon 550D camera in the darkroom. The extent of the diffusion of bone cement spray was noted during the experiments. According to the luminescent intensity shown on the drawing paper, the intensity decreased as the distance increased. The intensity of area wall within circle I was 72.6% ± 5.8%; within circle II, it was 22.1% ± 4.2%, and within circle III, it was 5.4% ± 1.5% (Fig. 2b). The intensity of the bottom area within circle I was 66.5% ± 6.1%; within circle II, it was 28.1% ± 4.8%, and within circle III, it was 5.4% ± 1.4% (Fig. 2c). The difference in intensity between each circle was significant. (*p* < 0.001) There was no difference in signal intensities, however, between the wall and bottom specimens without cover (Fig. 2d).

### Comparison between with cover and without cover

**Fig. 3:**
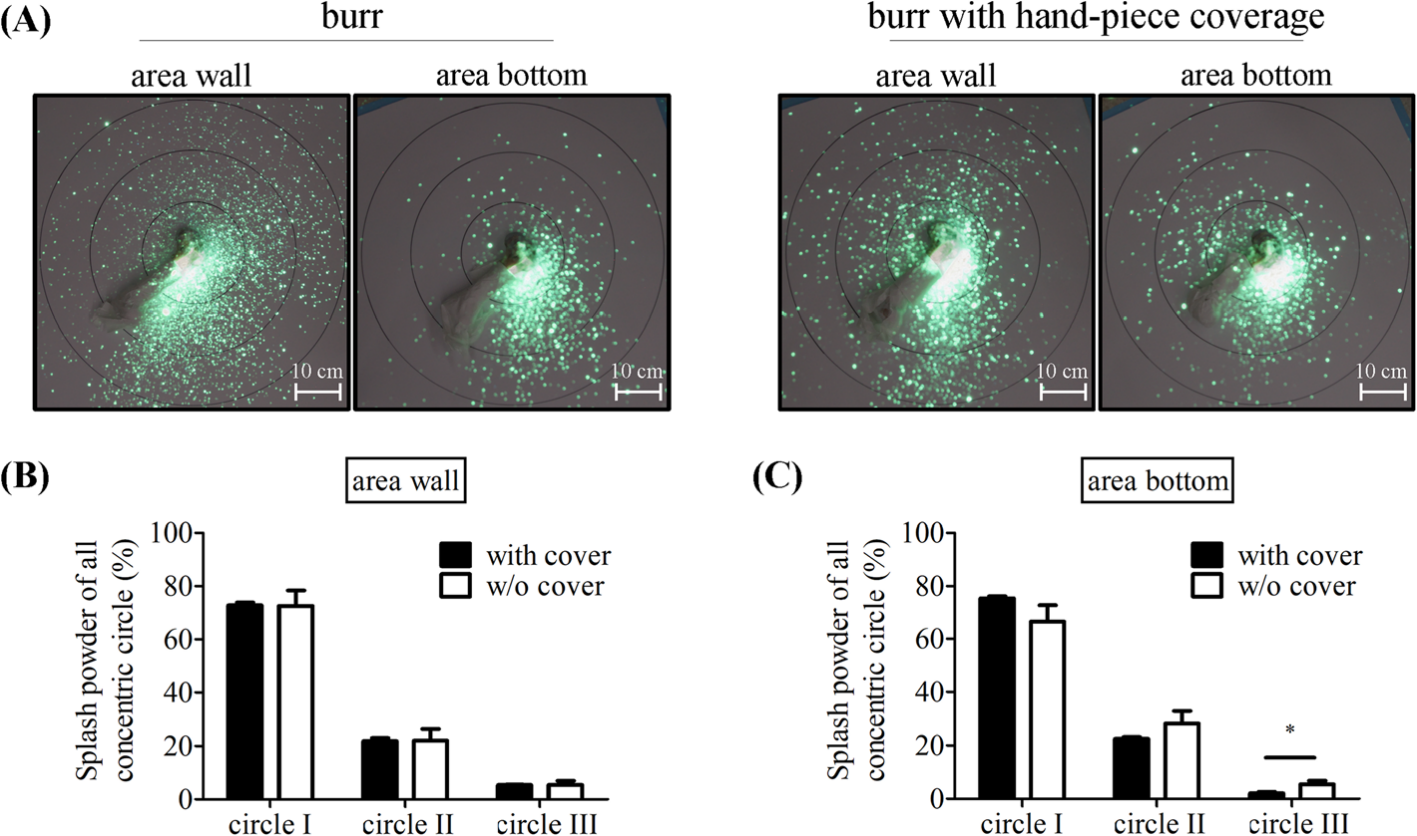
Comparison between with cover and without cover. **a** Comparison between without cover and coverage. **b** No difference when burring the wall **c** bone cement retained more in the 20 cm under coverage. (*p = m 0.018)

To compare the signals with and without cover, we analyzed the intensities of cement spread after burring the bottom area. The intensities of the bottom specimen decreased significantly after cover only in circle III. We recorded the following: circle I, 75.4% ± 0.8 and 66.5% ± 6.1% (*p* = 0.065); circle II, 22.5% ± 0.7, and 28.1% ± 4.8% (*p* = 0.114); and circle III, 2.1% ± 0.6 and 5.4% ± 1.4% (*p* = 0.018) (Fig. 3c). Meanwhile, the intensities of cement spread after burring the area wall were similar between the specimens with and without cover at any distance: circle I, 72.8% ± 1.1 and 72.6% ± 5.8% (*p* = 0.946); circle II,21.8% ± 1.2 and 22.1% ± 4.2% (*p* = 0.919); and circle III, 5.4 ± 0.3 and 5.4% ± 1.5% (*p* = 0.974) (Fig. 3b). Under the handpiece cover, the bone cement sprayed while burring the bottom area retained more in the 10-cm circle when burring the cement in the bottom of the cavity and sprayed less out of the 20-cm circle. Nevertheless, even with a cover, the high-speed burr still caused the bone cement to spray.

### Bone cement over the glove

**Fig. 4:**
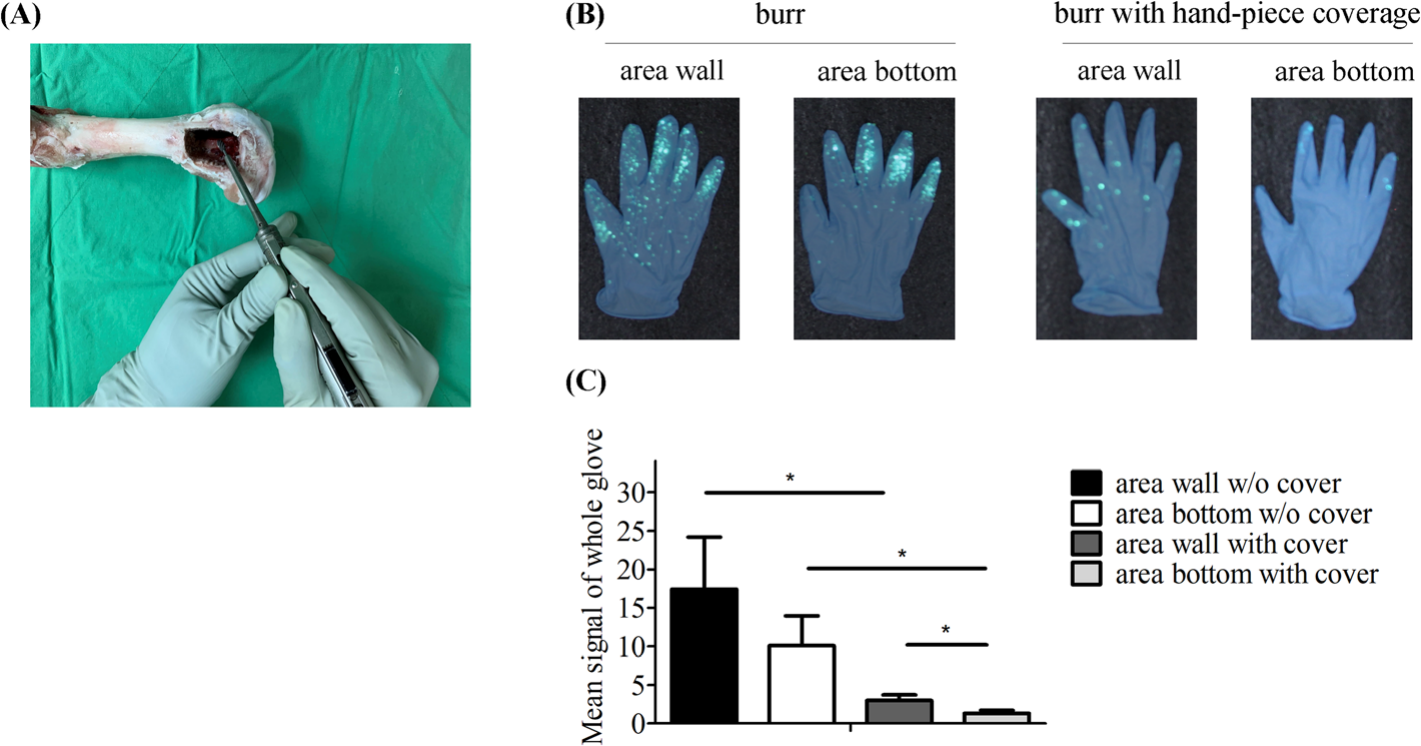
Bone cement over the glove. **a** The surgical demonstration **b** luminescent bone cement splash on the gloves, and **c** histogram of luminescent bone cement splash after burring the area wall and bottom, with or without coverage. *, *p* < 0.05

The third part of the experiment involved recording the amount of bone cement spray on the glove during burring. The intensity of the cement spray over the glove after burring the area wall was 17.40% ± 6.82% and the area bottom was 10.07% ± 3.90%, indicating that the luminescent bone cement sprayed widely over the glove during burring. The intensity of spray over the glove of the wall area with handpiece cover was 3.00% ± 0.71%, and the bottom area was 1.32% ± 0.39% (Fig. 4b). The signal intensity of the area wall and bottom decreased under the handpiece cover (wall: *p* = 0.022, and bottom: *p* = 0.018). Without the handpiece cover, there was no difference between the wall and the bottom (*p* = 0.181). However, the signal intensity on the glove when burring the area wall under the handpiece cover was greater than the intensities on the glove when burring the area bottom under the handpiece cover (*p* = 0.023) (Fig. 4c).

## Discussion

Adjuvant therapy by high-speed burr after intralesional curettage is a common surgical procedure. To evaluate the correlation between high-speed burring and local tumor seeding, we used luminescent bone cement to simulate the tumor cells. Surgical management of a local aggressive bone tumor, such as a GCT or low-grade chondrosarcoma, includes intralesional curettage or resection. When performing intralesional curettage, the use of a power burr to enlarge the cavity 1 cm to 2 cm in all directions is considered standard procedure [10]. However, aggressive bone tumors such as GCTs currently present a treatment challenge, due to local recurrence rates of 8 to 62% and metastatic rates of 1.5 to 7% [11].

Compared to wide excision, intralesional curettage offers an opportunity to preserve the joint and provides better postsurgical limb function. However, the recurrence rate is a concern when intralesional curettage is performed. Lausten et al. reported their results with 31 GCT cases treated between 1954 and 1987. Among 18 patients treated with intralesional curettage, 10 (56%) had local recurrences [12]. Malek et al. reported a study of 40 patients with long bone GCT treated with curettage, burring, bone grafting, and without any adjuvant treatment between 1997 and 2002. The local recurrence rate within the first 30 months of surgery was 32.5% [13]. Li et al. reported the outcome of 179 patients treated for GCT between 1998 and 2010. The local recurrence rate of intralesional curettage was 41.9% [14]. Thus, although the use of a high-speed burr is considered a standard procedure, the efficacy and the potential of contamination are still worth considering.

In order to explore the influence of the different intraosseous tumor locations, we divided the locations into intraosseous wall and intraosseous bottom. However, after comparing the area wall and area bottom, we found no differences in the intensity of the luminescent bone cement spray. Both areas had similar luminescent bone cement spread after burring, and both caused some degree of contamination. Therefore, the tumor seeding over surrounding areas that is caused by a high-speed burr will happen no matter where the burr is used. This type of tumor seeding may have contributed to potential local tumor recurrence.

The results demonstrated that around 66% of seeding tumor was located in the 10-cm circle of the surgical field where bone was burred. Between 10 cm and 20 cm, the proportion of seeding tumor was around 30%. Beyond 20 cm, the proportion of seeding tumor declined to 5%. The intensity decreased as the distance increased. According to the experiment results, adjuvant therapy by high-speed burr may cause tumor local seeding and local recurrence. Therefore, in order to reduce the chances of tumor spreading during operation, adequate protection and irrigation should be used routinely during high-speed burring. According to our experiments, the range of irrigation and suction should be as far as possible. The bone cement spray can be as far as 20 cm. Therefore, irrigation and suction is essential after high-speed burring to eradicate the bone tumor cell.

Another reason for local tumor seeding is contamination from the surgeon’s glove or other surgical instruments. In this study, we found luminescent bone cement not only in the surgical field but also sprayed over the surgical glove. A case report study showing a donor site over the anterosuperior iliac spine, representing metastasis from the distal tibia GCT, suggests that residual bone over the glove can cause contamination and implantation of tumor at the graft harvesting site [15]. To decrease the chances of recurrence due to bone seeding via the residual bone over the glove, surgeons should change their gloves frequently.

To prevent tumor seeding during burring, we wondered if using a handpiece cover over the burr could prevent tumor spread. In the section of the test area bottom, the luminescent bone cement spray in circle III decreased significantly after the handpiece was covered. However, this increased the spread of the luminescent bone cement within a 10-cm surgical field. This result suggests that handpiece coverage of the burr may prevent the tumor from spreading beyond 20 cm. However, it will also increase tumor spread closer than 10 cm. The intensity over the glove declined from 17 to 3% when burring the area of the wall (*p* = 0.022). Meanwhile, the intensity over the glove decreased from 10 to 1% when burring the area of the bottom (*p* = 0.018). This suggests that the coverage cannot prevent the luminescent bone cement spray from contaminating the surgical area. However, the use of coverage can reduce the luminescent bone cement spray on the glove.

A benign aggressive bone tumor such as a GCT can cause pathological fracture or soft tissue extension. The recurrence rate will increase [14]. When van der Heijden et al. reviewed a series of 48 patients with pathological fractures due to GCTs, the authors found that the recurrence rate was higher after curettage with adjuvant therapy when compared with resection alone (30% versus 0%, respectively). Recurrence risk appears higher with extension into soft tissue [16]. High-speed burring may break the intraosseous structure, which would eventually lead to pathological fracture. Meanwhile, the tumor spread to the surrounding area after high-speed burring can be regarded as a kind of soft tissue extension.

### Limitation

The study had several limitations. First, bone cement spray only represented tumor cells. The tumor cells could be extinguished by hyperthermic reaction when high-speed burring was applied. The tumor seeding is not directly evaluated. Second, the size and speed of the burr should have impact on the result. However, we chose one appropriate size of the burr and the maximum of the speed to test the range of the bone cement can spray. Thirdly, animal study is different to clinical practice. The High-speed burr is generally accepted as the standard and effective method to cure giant cell tumor. However, the study showed us the influence of High-speed burr to local surgical field. The relationship between bone spray and tumor seeding needs further investigation.

## Conclusions

High-speed burring can cause the bone cement to spray over the surgical field up to a 20-cm concentric circle from the surgical site. Meanwhile, the surgeon’s glove is also contaminated. The handpiece cover cannot reduce the intensities of the bone cement spray in the surgical area but can significantly decrease the bone cement spray over the glove. Although the bone cement cannot fully represent the tumor cell, but we can imagine the potential contamination due to high-speed burring through this study. Further examination is needed to investigate the correlation of the bone cement spray and tumor seeding.

## Data Availability

The datasets used and/or analyzed during the current study are available from the corresponding author on reasonable request.

## Abbreviations

GCT: Giant cell tumor
UV: Ultraviolet
